# A prospective study on ambulatory care provided by primary care pediatricians during influenza season

**DOI:** 10.1186/1824-7288-40-38

**Published:** 2014-04-23

**Authors:** Antonietta Giannattasio, Andrea Lo Vecchio, Carmen Napolitano, Laura Di Florio, Alfredo Guarino

**Affiliations:** 1 Medicine and Health Sciences Department, University of Molise, c/da Tappino 86100, Campobasso, Italy; 2 Department of Translational Medical Sciences, University Federico II, Naples, Italy

**Keywords:** Primary care pediatrician, Diagnosis, Prescription, Flu-like illness, Children

## Abstract

Aim of this study was to obtain a picture of the nature of the primary care pediatricians’ visits during a winter season. We investigated reasons for visits, diagnosis, and pattern of prescription in 284 children. The reason for visit was a planned visit in 54% of cases, a well-being examination in 26%, and an urgent visit for an acute problem in 20% of cases. Cough was the most common symptom reported (61%). The most common pediatricians’ diagnosis was flu-like syndrome (47%). No disease was found by pediatrician in 27% of children with a symptom reported by caregivers. Antibiotics were prescribed in 25% of children, the vast majority of which affected by viral respiratory infections. The unjustified access to physician’s visit may lead to a inappropriate prescription of drugs.

## Introduction

Primary ambulatory health care is important for maintaining good health in individuals by providing preventive interventions and early treatment of illness [1]. Children access the primary care health system for three main reasons: preventive care, illness care, and trauma or injury [1]. A progressive increase in ambulatory care visits has been reported in the last years in U.S. [2,3]. The global physicians’ activity is loaded with important day-of-the-week and seasonal variations [4]. During the winter, the annual outbreak of influenza leads to a high rate of outpatient visits and antibiotic prescription in children [5-10]. Understanding the pattern of visits at primary care pediatricians (PCPs) offices is important to develop policies aimed to improve access to health services and quality of care provided by physicians.

Aim of this prospective study was to obtain a picture of the nature of the PCPs visits during a winter season. The major reasons for visits, pattern of diagnosis, impact of flu-like illness on PCPs activity, and pattern of prescriptions are analysed.

## Methods

The survey was conducted during an 8-week winter period (December-January) in three PCPs practices in Naples (Campania Region, Italy) in 2011–2012 season. The PCPs enrolled in the study, who have similar age, year of specialization and number of assigned patients, served globally 2751 children aged 0–14 years. A sample of consecutive children was included. Data were prospectively collected by a pediatric nurse through direct observation during PCPs’ visits. The nurse visited the PCPs offices 3 times a week for each paediatrician, for a total of 72 working shifts. All children observed in the index days were enrolled and data were collected through a standardized form including patient demographic information, clinical data, time to visit, type of visit, patient’s principal reason for visit, physician diagnosis, and prescriptions. The research was conducted according to the Helsinki declaration, approval by ethical committee was not formally required being an observational study. However, the informed consent was obtained by legal guardians of enrolled children.

Time to visit was defined as the period between the onset of symptoms and the medical visit. It was classified as: urgent visit (a visit for an acute onset problem or for an exacerbation of a chronic problem), planned visit (a visit for a deferred symptom, check for a chronic conditions, or immunization), visit for child well-being examination included in the PCPs’ routine activity. The main reason for visit reflects caregivers’ perspective of the major reason for seeing care, and it may differs from final physician’s diagnosis.

Criteria for diagnosis of influenza like illness (ILI) were the sudden onset of fever, with respiratory and/or systemic symptoms during the influenza season [4,11]. Definition of upper respiratory tract infection (URTI) was based on a single symptom or on the presence of a cohort of different respiratory symptoms, irrespective of season and presence of fever.

## Results

**Figure 1:**
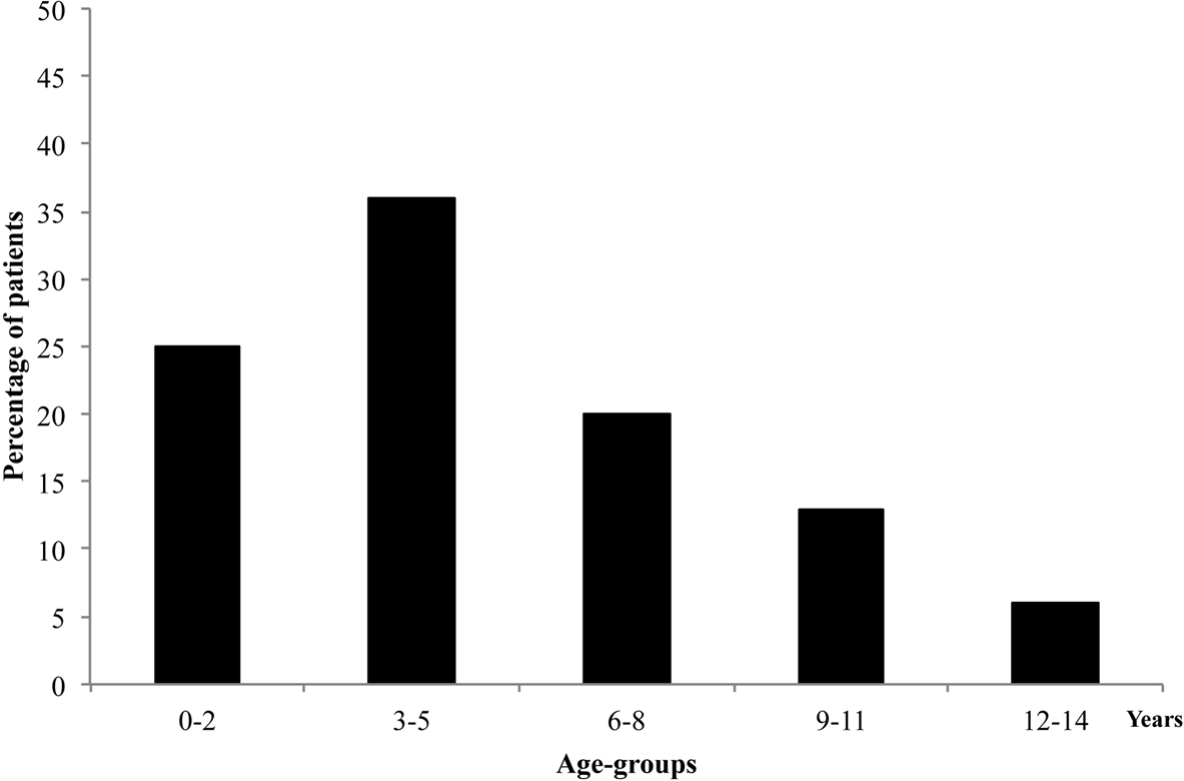
Distribution of 284 enrolled children according to age-groups.

**Figure 2:**
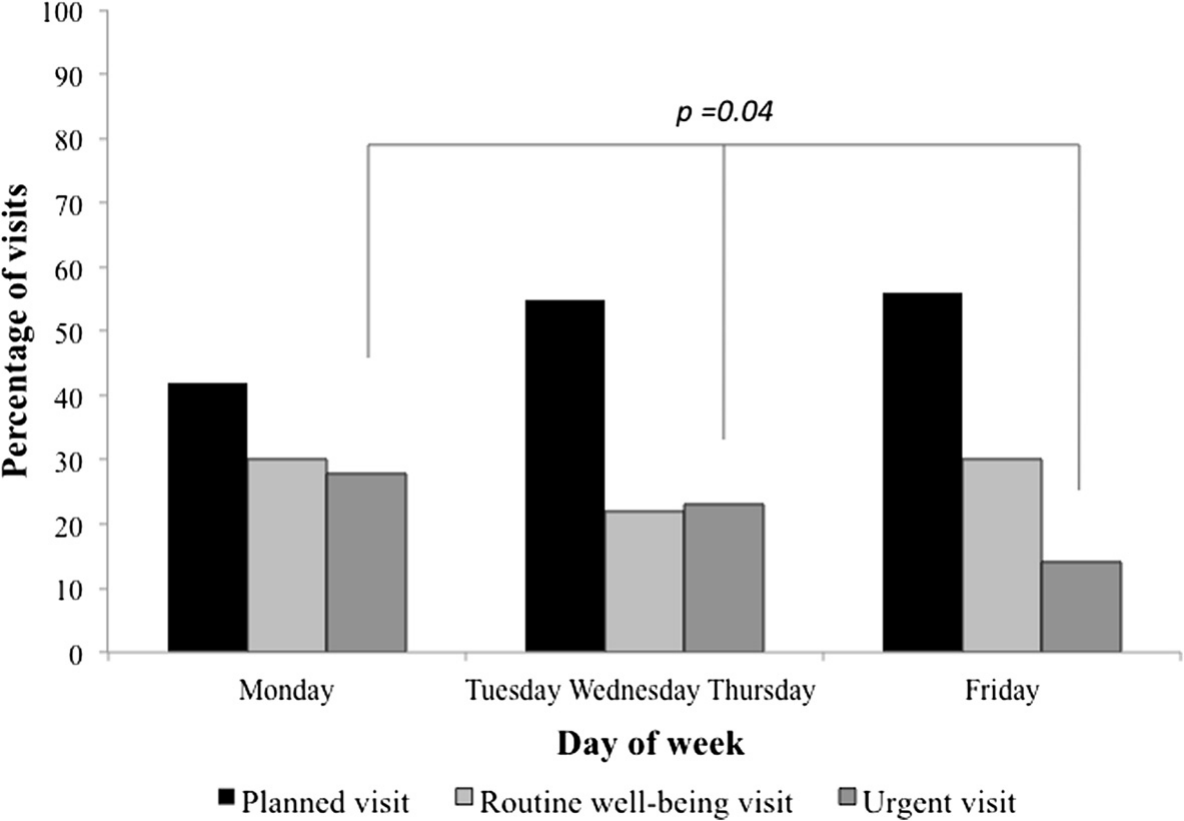
Type of visit according to the day of week.

A total of 284 children (131 males; mean age 4.8 ± 3.7 years; 12% with an underlying chronic conditions) were enrolled (Figure 1). The type of visit was: a planned visit in 153/284 (54%) cases, a well-being examination in 75 (26%) and an urgent visit for an acute problem in 56 (20%). A higher percentage of urgent visits were recorded on Monday (Figure 2).

**Figure 3:**
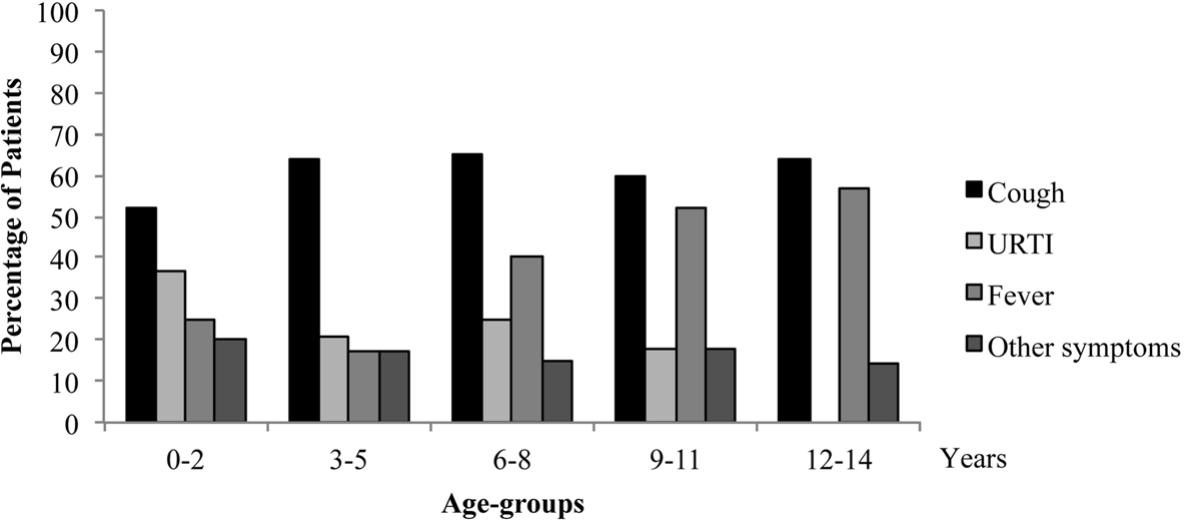
Patient’s principal reason for visit according to age groups in 188 children presenting with clinical symptoms.

Caregivers required a visit because of symptoms in 188/284 (66%) children. Specifically, all 56 caregivers requiring an urgent visit reported the presence of one or more symptoms. Other 132 children presenting symptoms were seen during planned visits. Cough was the most common symptom in all age-groups (115/188, 61%), followed by fever (80, 43%), upper respiratory symptoms (44, 23%), and other symptoms such as abdominal pain, headache, vomit (33, 17%). The symptom distribution showed an age-related pattern, with upper respiratory tract symptoms more frequent in children aged 0–2 years (38%), compared to the other groups (p < 0.001) (Figure 3).

Among 209 children receiving a planned or an urgent visits, 137 (73%) had a final diagnosis by PCPs of an acute disease. Specifically, in 51 out of 188 (27%) children whose caregivers have required a visit because of presence of symptoms, no disease at medical examination was found by PCPs. The most common diagnoses made by PCPs were ILI (65 cases, 47%), URTI (40 cases, 29%), pneumonia (5 cases, 4%).

**Figure 4:**
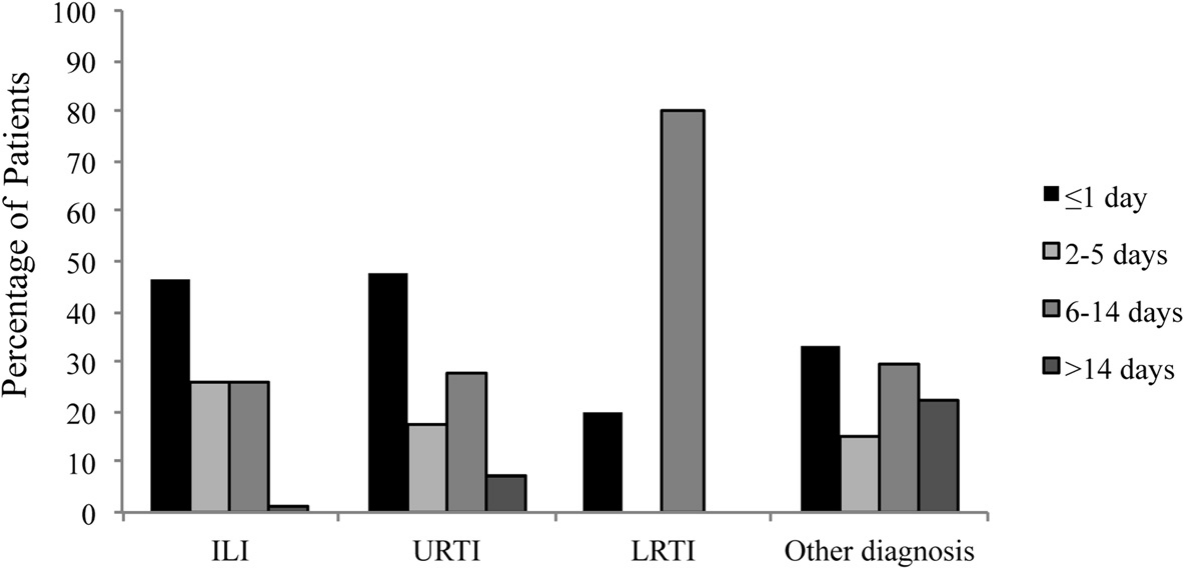
**Timing of requiring a medical visit since on the onset of symptoms according to the final diagnosis in 137 children with a pediatrician diagnosis of acute disease.** ILI: Influenza-like illness; URTI: upper respiratory tract infection; LRTI: lower respiratory tract infection.

Time to visit was significantly related to the final diagnosis. Caregivers of children with a final diagnosis of ILI or URTI required more frequently a visit within one day from the onset of symptoms compared to the groups with lower respiratory tract infections or other diagnosis (p = 0.025) (Figure 4).

**Figure 5:**
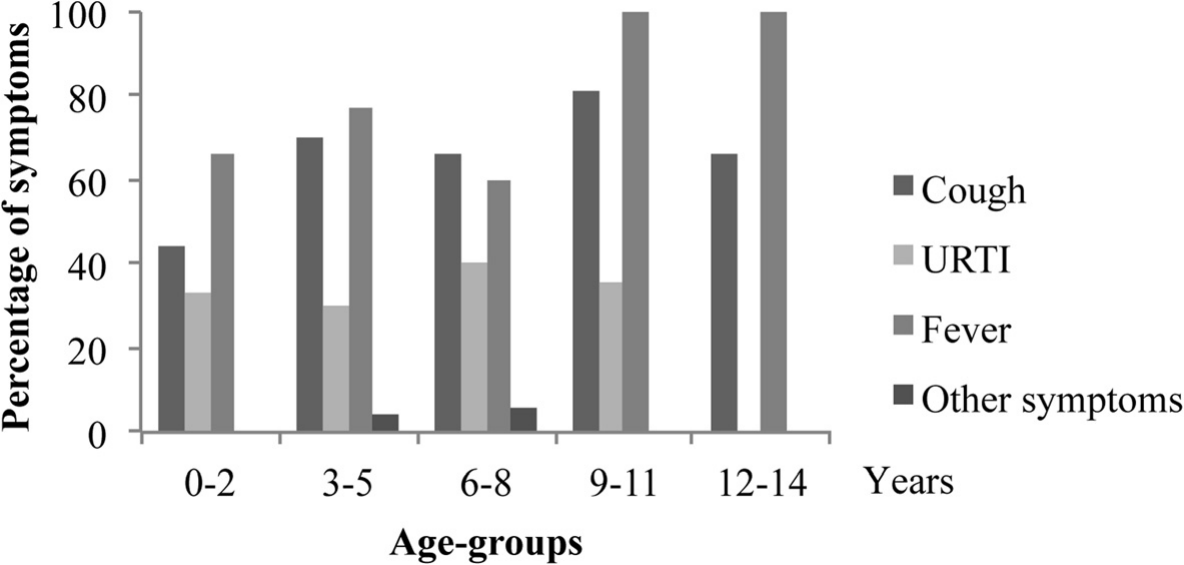
**Distribution of symptoms in 65 children with a diagnosis of ILI according to the age groups.** “Other symptoms” category includes: headache, earache, sore throat, abdominal pain, diarrhea, vomit. URTI: upper respiratory tract infection.

In the group of 65 children (mean age 5.7 ± 3.4 years) receiving a diagnosis of ILI, at the time of PCPs visit the most common symptom was fever (78%), followed by cough (68%) and other upper respiratory tract symptoms (32%), with no difference in age distribution (Figure 5).

As additional data, only 5% of enrolled children (and only one of 33 children with an underlying chronic condition) had already received or were going to receive seasonal influenza vaccination.

One or more medications were prescribed to 96/137 (70%) children with a final diagnosis made by PCPs. Anti-inflammatory drugs were prescribed alone in 62 (65%) cases, antibiotics in 22 (23%) cases, and both medications in 12 (12%) children. Potential determinants of antibiotic prescription were child age and duration of symptoms. A trend toward higher prescription in younger children was registered, although not statistically significant (p = 0.1). A duration of symptoms of 6–13 days was more frequently associated with antibiotic prescription (44% of children in group receiving antibiotics versus 26% not receiving antibiotics; p = 0.04). The diagnosis in 34 children receiving antibiotics were: ILI in 13 (38%) cases, URTI in 12 (35%) cases, pneumonia in 5 (15%) and “other diagnosis” in 4 (12%) cases. According to the presence of an underlying chronic disease, a trend in lower prescription of antibiotics in at-risk children compared to otherwise healthy children was registered (17% versus 24%, p > 0.05).

In the group of 65 children with ILI, anti-pyretics were prescribed in 47 (72%) cases, antibiotics (alone or together with anti-pyretic) in 13 cases (20%), and other medications in 11 (17%) cases. The most common reasons for antibiotic prescription were: persistence of respiratory symptoms without improvement for >5 days (3 children), clinical signs of pneumonia (2 cases), fever lasting >3 days (3 cases), and self-prescription by parents (5 children). PCPs required laboratory (3 cases) or radiological investigations (1 case) in few cases.

## Discussion

Ambulatory health care in the U.S. has been examined by analyzing data from multiple settings [2,12]. About 20% of all ambulatory visits in 2007 in the U.S. were due to non illness conditions, such as routine check-up [3]. Preventive care is incorporated into pediatric practice in order to promote health for individual children and adolescents and it is a frequent reason for ambulatory visits, accounting for 20-30% of them [13,14]. As a consequence, PCPs spent a considerable time and efforts for non urgent visits, with a possible decreased time to visit more serious patients in their practice. Annually, during influenza season, an increase in the activity at PCPs office is observed [9,10] and the high number of office-based preventive care interventions may exceed the capacity of the health care system to fulfil the requests for acute conditions. Such an increase of the need of medical visits was confirmed in our study. A major burden on childhood health care request may be more frequent during an influenza pandemic [15-17]. During the last 2009 H1N1 pandemic, the highest consultation rate and hospitalization for ILI were recorded in children [15,18]. It can be hypothesized that the inappropriate overflow of health care services registered was due, at least in part, to the anxiety and concern by the population for H1N1 influenza, also because of extensive media campaigns, rather than with a real increased incidence or severity of ILI. However, a major burden of requests may occur independently of specific concern. To confirm the inappropriate request for a medical visit by caregivers, our data showed that more than 20% of children who were visited at PCPs had no disease as final diagnosis and no real need for medical consultation.

Respiratory tract diseases were the most common diagnosis, accounting for about 37% of all visits, and for more than 70% of children with a final diagnosis of an acute disease. It is to note that in our study, the number of ILI cases was based on practitioner’s medical visit, providing a more reliable information compared to previous studies [15,17].

In this survey, only 5% of enrolled children had received seasonal influenza vaccination. Although in Italy seasonal influenza vaccine is recommended and offered free of charge to children with chronic conditions, flu vaccination rates are very low in almost all pediatric at-risk categories [19-23]. Indeed, in our population there was only one vaccinated child out of 33 children that should have been immunized because of a chronic condition.

The high rate of antibiotic prescription deserves a specific comment. Antibiotics are still commonly prescribed for children with conditions for which they provide no benefit, including respiratory infections whose etiology is likely a viral infection [24-26]. As much as 26% of visits of children aged <5 years resulted in an antibiotic prescription in office-based settings, compared to 32% in those emergency departments [27]. It has been reported that in Italian southern regions antibiotics are more frequently prescribed that in the northern and central regions [28,29]. In the present study, antibiotics were prescribed in 25% of children with a diagnosis of illness made by PCPs. However, antibiotics were inappropriately prescribed for viral infections (ILI or URTI) in more than 70% of cases. Although not statistically significant, at-risk children received less antibiotics than “otherwise healthy” children. This may be related to the presence of other long-lasting treatments, or in some cases to the consultation with referral institution for chronic diseases (not rare for chronic patients).

In conclusion, based on our data, there is a clear need to limit the unjustified access to physician’s visit that may lead to a consequent inappropriate prescription of drugs in addition to costs for health care system and families. An approach based on a telephone triage by PCPs might reduce the inappropriate access to their offices, and the excess of non medical access to the emergency department. Moreover, a telephone triage could provide information and advice to patients, and could help in optimizing the PCPs activities, with specific initiatives such as postponing the well-being visits. During a period of high burden of infectious disease it is necessary to develop strategies in order better allocate health care resources and ensure a high quality of care provided by primary care physicians.

## Abbreviations

PCPs: Primary care pediatricians; SD: Standard deviation; ILI: Influenza-like illness; URTI: Upper respiratory tract infection.

## Competing interests

The authors declare that they have no competing interests.

## Authors’ contributions

AG coordinated the study, performed statistical analysis and drafted the manuscript. ALV helped to coordinate the study and draft the manuscript. CN and LDF created the database for analysis of data and contributed to the first draft of the manuscript. AG conceived the study, and participated in its design and supervision. All authors read and approved the final manuscript.

## References

[B1] Freid VM Makuc DM Rooks RN Ambulatory health care visits by children: principal diagnosis and place of visit Vital Health Stat 13 1998 137 1 23 9631643

[B2] Schappert SM Burt CW Ambulatory care visits to physician offices, hospital outpatient departments, and emergency departments: United States, 2001–02 Vital Health Stat 13 2006 159 1 66 16471269

[B3] Schappert SM Rechtsteiner EA Ambulatory medical care utilization estimates for 2007 Vital Health Stat 13 2011 169 1 38 21614897

[B4] Flamand C Larrieu S Couvy F Jouves B Josseran L Filleul L Validation of a syndromic surveillance system using a general practitioner house calls network, Bordeaux, France Euro Surveill 2008 19 25 13 18761939

[B5] Ge Y Cai J Wang X Yao W Shen J Zhu Q Wang X Zeng M Childhood influenza in the outpatient setting in Shanghai, China Pediatr Infect Dis 2012 31 e111 e116 10.1097/INF.0b013e318257172d 22481425

[B6] Centers for Disease Control and Prevention (CDC) Update: influenza activity - United States, 2011–12 season and composition of the 2012–13 influenza vaccine MMWR Morb Mortal Wkly Rep 2012 61 414 420 22672977

[B7] Centers for Disease Control and Prevention (CDC) Update: influenza activity - United States, September 30, 2012-February 9, 2013 MMWR Morb Mortal Wkly Rep 2013 62 124 130 23425961 PMC4604885

[B8] Tsolia MN Logotheti I Papadopoulos NG Mavrikou M Spyridis NP Drossatou P Kafetzis D Konstantopoulos A Outpatient flu study group. Impact of influenza infection in healthy children examined as outpatients and their families Vaccine 2006 24 5970 5976 10.1016/j.vaccine.2006.05.006 16759761

[B9] Izurieta HS Thompson WW Kramarz P Shay DK Davis RL De Stefano F Black S Shinefield H Fukuda K Influenza and the rates of hospitalization for respiratory disease among infants and young children N Engl J Med 2000 342 232 239 10.1056/NEJM200001273420402 10648764

[B10] Marchisio P Baggi E Bianchini S Principi N Esposito S Clinical and socioeconomic impact of pediatric seasonal and pandemic influenza Hum Vaccin Immunother 2012 8 17 20 10.4161/hv.8.1.18145 22252002

[B11] Martirosyan L Paget WJ Jorgensen P Brown CS Meerhoff TJ Pereyaslov D Mott JA EuroFlugroup The community impact of the 2009 influenza pandemic in the WHO European region: a comparison with historical seasonal data from 28 countries BMC Infect Dis 2012 12 36 10.1186/1471-2334-12-36 22325082 PMC3292513

[B12] Forrest CB Whelan EM Primary care safety-net delivery sites in the United States: a comparison of community health centers, hospital outpatient departments, and physicians’ offices JAMA 2000 284 2077 2083 10.1001/jama.284.16.2077 11042756

[B13] Olson LM Inkelas M Halfon N Schuster MA O’Connor KG Mistry R Overview of the content of health supervision for young children: reports from parents and pediatricians Pediatr 2004 113 1907 1916 15173461

[B14] Cherry DK Burt CW Woodwell DA National ambulatory medical care survey: 2001 summary Adv Data 2003 337 1 44 12924075

[B15] Van Cauteren D Vaux S de Valk H Le Strat Y Vaillant V Lévy-Bruhl D Burden of influenza, healthcare seeking behaviour and hygiene measures during the A(H1N1)2009 pandemic in France: a population based study BMC Public Health 2012 12 947 10.1186/1471-2458-12-947 23127166 PMC3508974

[B16] Carrat F Sahler C Rogez S Leruez-Ville M Freymuth F Le Gales C Bungener M Housset B Nicolas M Rouzioux C Influenza burden of illness: estimates from a national prospective survey of household contacts in France Arch Intern Med 2002 162 1842 1848 10.1001/archinte.162.16.1842 12196082

[B17] Brooks-Pollock E Tilston N Edmunds WJ Eames KT Using an online survey of healthcare-seeking behaviour to estimate the magnitude and severity of the 2009 H1N1v influenza epidemic in England BMC Infect Dis 2011 11 68 10.1186/1471-2334-11-68 21410965 PMC3073914

[B18] Giannattasio A Lo Vecchio A Russo MT Pirozzi MR Barbarino A Ruberto E Campa A Guarino A Pandemic flu: a comparative evaluation of clinical, laboratory, and radiographic findings in HIV-positive and negative children AIDS 2010 24 2292 2294 10.1097/QAD.0b013e32833d2096 20639725

[B19] Piano nazionale prevenzione vaccinale 2012–2014 Available at: http://www.salute.gov.it/imgs/C_17_pubblicazioni_1721_allegato.pdf 23532166

[B20] Pandolfi E Marino MG Carloni E Romano M Gesualdo F Borgia P Carloni R Guarino A Giannattasio A Tozzi AE The effect of physician’s recommendation on seasonal influenza immunization in children with chronic diseases BMC Public Health 2012 12 984 10.1186/1471-2458-12-984 23153092 PMC3585468

[B21] Pandolfi E Carloni E Marino MG Atti ML C d Gesualdo F Romano M Giannattasio A Guarino A Carloni R Borgia P Volpe E Perrelli F Pizzuti R Tozzi AE Immunization coverage and timeliness of vaccination in Italian children with chronic diseases Vaccine 2012 30 5172 5178 10.1016/j.vaccine.2011.02.099 21414380

[B22] Giannattasio A Lo Vecchio A Franzese A Prisco F Femiano P Guarino A Redundancy of roles by physicians in charge of paediatric diabetes is a barrier to flu immunisation Arch Dis Child 2010 95 399 400 10.1136/adc.2010.182980 20457709

[B23] Giannattasio A Squeglia V Lo Vecchio A Russo MT Barbarino A Carlomagno R Guarino A Pneumococcal and influenza vaccination rates and their determinants in children with chronic medical conditions Ital J Pediatr 2010 36 28 10.1186/1824-7288-36-28 20346141 PMC2851708

[B24] Hersh AL Shapiro DJ Pavia AT Shah SS Antibiotic prescribing in ambulatory pediatrics in the United States Pediatr 2011 128 1053 1061 10.1542/peds.2011-1337 22065263

[B25] Atti ML C d Massari M Bella A Boccia D Filia A Salmaso S SPES study group Clinical, social and relational determinants of paediatric ambulatory drug prescriptions due to respiratory tract infections in Italy Eur J Clin Pharmacol 2006 62 1055 1064 10.1007/s00228-006-0198-8 17021889

[B26] Osservatorio ARNO bambini. I profili assistenziali delle popolazioni in età pediatrica. Rapporto 2011 Available at: http://sip.it/wp-content/uploads/2011/10/ARNO_bambini_rapporto_2011.pdf

[B27] Halasa NB Griffin MR Zhu Y Edwards KM Differences in antibiotic prescribing patterns for children younger than five years in the three major outpatient settings J Pediatr 2004 144 200 205 10.1016/j.jpeds.2003.10.053 14760262

[B28] Piovani D Clavenna A Bonati M Interregional Italian Drug Utilisation Group Drug use profile in outpatient children and adolescents in different Italian regions BMC Pediatr 2013 13 46 10.1186/1471-2431-13-46 23557352 PMC3623731

[B29] Piovani D Clavenna A Cartabia M Bonati M Antibiotic Collaborative Group The regional profile of antibiotic prescriptions in Italian outpatient children Eur J Clin Pharmacol 2012 68 997 1005 10.1007/s00228-011-1204-3 22271296

